# Donation After Circulatory Death following Withdrawal of Life-Sustaining Treatments. Are We Ready to Break the Dead Donor Rule?

**DOI:** 10.1007/s11673-024-10382-8

**Published:** 2024-09-05

**Authors:** Sara Patuzzo Manzati, Antonella Galeone, Francesco Onorati, Giovanni Battista Luciani

**Affiliations:** 1 Department of Surgery, Dentistry, Pediatrics and Gynecology, Division of History of Medicine and Bioethics, University of Verona, Piazzale A. Stefani 1, 37129 Verona, Italy; 2 Department of Surgery, Dentistry, Pediatrics and Gynecology, Division of Cardiac Surgery, University of Verona, Piazzale A. Stefani 1, 37129 Verona, Italia

**Keywords:** Donation after circulatory death, Dead donor rule, Withdrawal of Life-Sustaining Treatment, No-touch period, Maastricht III category, Organ procurement

## Abstract

A fundamental criterion considered essential to deem the procedure of vital organ procurement for transplantation ethical is that the donor must be dead, as per the Dead Donor Rule (DDR). In the case of Donation after Circulatory Death (DCD), is the donor genuinely dead? The main aim of this article is to clarify this uncertainty, which primarily arises from the fact that in DCD, death is determined based on cardiac criteria (Circulatory Death, CD), rather than neurological criteria (Brain Death, BD), and that to allow the procurement procedure, physicians reperfuse the organs in an assisted manner. To ensure that the cessation of circulation leads to the irreversible loss of brain functions, DCD regulations require that physicians wait a certain period after CD before commencing vital organ procurement. However, during this “no-touch period,” the organs are at risk of damage, potentially rendering them unsuitable for transplantation. When DCD is performed on patients whose CD follows a Withdrawal of Life-Sustaining Treatment (WLST) (DCD Maastricht III category), how long should the no-touch period last? Does its existence really make sense? Does beginning the procedure of vital organ procurement immediately after WLST constitute a violation of the DDR that can be ethically justified? The discussion aims to provide arguments in support of the non-absoluteness of the DDR.

## Introduction

The procedure of organ transplantation necessitates the surgical procurement of organs from one individual and their transplantation into another. For this procedure to be ethically justified, our societies generally adhere to the following criteria.

1. In instances where vital organs are removed, the individual must be deceased at the time of procurement to ensure that the procedure does not precipitate their demise (Arnold et al. 1993; Entwistle et al. 2022; Truog et al. 2013).
2. The objective of the transplantation should be to enhance both the life expectancy and quality of life of individuals suffering from terminal organ failure.
3. The decision to donate should have been made and expressed by the individual themselves during their lifetime, ensuring that the transplantation respects the donor’s expressed will (Shaw et al.  2020).
4. Organs must be donated without any financial or material compensation, upholding the thesis that the body and its parts are not commodifiable and affirming that the distribution of organs should not be contingent upon the recipient’s financial capacity (Alpinar-Şencan et al. 2017).

As it is well-established, the first criterion is also referred to as the Dead Donor Rule (DDR) (Robertson 1999). As is often the case in ethics, both the individual four criteria and the DDR can be, and indeed are, subjects of debate. In this study, we will focus our discussion on the last criterion in relation to a specific case of transplantation: that of Donation after Circulatory Death (DCD) after the Withdrawal of Life-Sustaining Treatment (WLST).

Early transplant programmes (DeVita et al. 1993), including the first heart transplant in 1967 (Barnard 1967), developed using organs from donors declared dead using circulatory criteria or non-heart-beating donors. Following the first publication of the definition of Brain Death (BD) and the criteria to ascertain BD by the Harvard Medical School in 1968 (Beecher 1968), most organ transplants have been performed with organs from donors declared dead using neurological criteria or heart beating donors. Organ shortage has led to the use of expanded donor criteria (Galeone et al. 2020) and has also resumed interest in DCD in addition to Donation after Brain Death (DBD). DCD transplant programmes are increasing in many countries accounting for about 20 per cent of overall organ donation in Europe (Lomero et al. 2020). Currently, more than 60 per cent of deceased donors in the Netherlands and more than 40 per cent in the United Kingdom are DCD (Eurotransplant International Foundation 2021; NHS Blood and Transplant 2021/2022). Recent studies showed that the adoption of a DCD heart transplant programme could potentially provide an increase in adult heart transplantation volume by approximately 15–30 per cent (Noterdaeme et al. 2013; Messer et al. 2015).

DCD donors can be classified into four categories according to the Maastricht classification of 1994 (Kootstra^1995^), subsequently modified in 2013 (6th International Conference on Organ Donation after Circulatory Death 2013). A fifth category also exists in countries where euthanasia is legal, and it includes patients who grant access to medically assisted circulatory death (Evrard 2014). The third category, called “Maastricht III,” in medical practice includes patients with catastrophic cerebral lesions and poor prognosis that do not fulfil all the neurological criteria for BD diagnosis. In these patients, a WLST is planned.

Afterwards, accordingly to patient’s and/or family wish, a proposal of organ donation could be discussed.

WSLT is usually followed by Cardiac Arrest (CA). Time elapsed from WSLT (dying patient) to asystole (dead patient) is called agonal phase and may have variable duration. If CA does not occur within a defined length of time following WLST, typically ninety minutes, the donation process is stopped, because during this phase thoraco-abdominal organs are subjected to a warm ischemic time that may damage the organs and compromise their quality.

After CA occurs it is necessary to observe a no-touch period to ensure that autoresuscitation will not occur (Smith et al. 2019). The no-touch period varies from two to thirty minutes across European countries (Lomero et al. 2020), with most protocols recommending five minutes of observation of apnoea and pulselessness before organ recovery may begin (Zorko et al. 2023). In Italy the no-touch period is twenty minutes, because the declaration of death with cardiac criteria requires continuous electrocardiographic recording showing the absence of any cardiac electrical activity for at least twenty minutes. In addition, the no-touch period must start after the establishment of cardiac electrical silence, rather than after circulatory arrest (absence of pulse regardless of the cardiac rhythm) (Italian Law n. 578/1993, artt. 1,2; Italian Decree n.582/1994). Both these requirements may significantly prolong the ischemic time during DCD and further damage organs. In many European countries (Lomero et al. 2020) and in the United States (Schroder et al. 2023) the pronouncement of death follows established practice, where the absence of a pulse verifies circulatory arrest. Mechanical asystole on an intra-arterial line is mostly used in practice to define this point. Electrical asystole is not required for the declaration of death and typically occurs several minutes later (Schroder et al. 2023).

After the declaration of death, it is possible to initiate all the procedures aimed at the recovery of the abdominal and thoracic organs. One technique to restore organs’ perfusion is the utilization of an extra-corporeal membrane oxygenation (ECMO) through femoral vessels cannulation, while the descending aorta is occluded with a balloon to prevent the perfusion of the brain. This preservation strategy is called Normothermic Regional Perfusion (NRP) (van de Leemkolk et al. 2020) and allows a better assessment of the organ function and a higher rate of organs procurement. While in the DBD donor organs are constantly perfused until procurement and subsequently protected with organ preservation solutions, in DCD donor organs undergo a variable time of inadequate perfusion and oxygenation that may significantly affect early and long-term results of transplantation, because of different susceptibilities to ischemia–reperfusion injury of each organ (Levvey et al. 2019; Dhital et al. 2020). Recent evidence suggests that there is no difference in short and long-term outcomes between DCD and DBD donors in solid organ transplantation, however several organ-specific DCD related complications do occur (Siddiqui et al. 2022; Ahmed et al. 2023).

Patients have the right to refuse treatment, even if it is life-sustaining, knowing that they will die, and to donate their organs if they wish. Following the WLST, the patient is so close to death, and will die regardless, and waiting even a few additional minutes could harm the organs and provide fewer benefits to others while not fully honouring the donor’s wishes. Can we then proceed with the removal of vital organs immediately after WLST, thereby hastening the patient’s death and thus violating the DDR? Should patient autonomy and beneficence towards others take precedence over the strict prohibition of causing their death? In the discussion, we will argue in support of this position.

## Discussion

The ethical criterion on the death of the donor is not centred around the question “What is death?” but rather “When does a person die?” It is generally believed that death occurs when life has ceased. However, the challenge arises from the fact that life and death are philosophical concepts and, as such, are not universally definable (Malpas et al. 1998). On the other hand, the medical community needs to standardize its procedures and to do this, it requires defining both death and the onset of life (Youngner et al. 1999). These are conventional definitions, susceptible to change over history as scientific progress enhances and refines our understanding of biological mechanisms. For example, while in ancient times death was conceived in terms of cessation of movement or body decomposition, in modern times it has been associated with the permanent and irreversible cessation of vital functions, leading to the definition of “Cardiac Death” or, when referring to functional aspects, “Circulatory Death” (CD) (Bernat 2013). However, in the Fifties and Sixties of the last century, the heart became a “resuscitable” and transplantable organ, leading to a new definition of death, that is BD (Beecher 1968). In Italy, to determine BD and thus declare a person’s death, besides the method that instrumentally verifies the irreversible cessation of all brain functions, another is based on the following statement: “Death due to cardiac arrest is considered to have occurred when respiration and circulation have ceased for a period of time that entails the irreversible loss of all brain functions” (Italian Law n. 578/1993, artt. 1,2). The time deemed sufficient to ensure the irreversible loss of brain functions is twenty minutes. Although a clinical verification of this loss is not conducted, after this period under Italian law the individual is considered and thus declared dead.

In the context of organ transplantation, if organ retrieval occurs after death has been determined with the neurological criterion, the procedure is termed DBD. If death has been determined with the second circulatory criterion, it is termed DCD. The terms DBD and DCD can lead to confusion and misunderstandings, instilling doubts that in the case of DCD, the person from whom vital organs are being extracted is not yet dead as they are not yet in BD, thereby violating the moral imperative “Do not kill” (a person) to “prey upon” the organs (Committee on Bioethics 2013; Jericho 2019; Nielsen Busch et al. 2022). In reality, in Italy as it stands, even in DCD, BD is considered to have actually occurred, it has just been determined “indirectly”, i.e. by waiting for a certain period after CD (twenty minutes). In summary, there is but one death, only that there are two criteria to determine it—“unifying concept of death” (White 2019). However, in ethical debate, we can include the perspective that the moral prohibition against killing a person is not inherently absolute (“ab-solutus,” freed from constraints, such irrespective of the specific context) and universal (applicable to all, always, and everywhere) but rather relative and as such admits exceptions. An example of an exception to the prohibition “Do not kill” in the healthcare context is Medically Assisted Death (MAD), whether it be Medically Assisted Suicide (MAS) or euthanasia, where under certain conditions, a physician either prescribes or administers a lethal drug to a patient at their request. On one hand, the conventional nature of the concept of death, and on the other, the non-absoluteness and universality of the moral prohibition “Do not kill,” in ethical debate open up the possibility of reflecting on the non-absoluteness and universality of the DDR. Additionally, it is also true that from the perspective of Italy, where, as mentioned, the no-touch period is tenty minutes and only after this duration a person is considered deceased, the practice of DCD in a country where this period is shorter, could constitutes a violation of the DDR.

Another misunderstanding that needs clarification pertains to the issue of NRP. When physicians proceed with DCD after waiting for the “x” period of time and the declaration of death, they restore circulation in an assisted manner to perfuse the organs and protect them from warm ischemia (Entwistle et al. 2022). This would constitute a violation of the DDR (Ross 2023). Indeed, it is highlighted that after CD, not only is the patient not necessarily dead, but they might also be revived through certain resuscitation efforts or by using extracorporeal circulation. Moreover, since oxygenated blood continues to flow to some vital organs, declaring death based on circulatory criteria might no longer be valid. To avoid violations of the DDR, death should be determined on neurological grounds (Entwistle et al. 2022). Upon closer examination, even though circulation is still present (assisted) when vital organs are removed, it does not imply that the person is still alive, as the “x” period of time has indeed passed. This period, as mentioned, in Italy is designed specifically to wait until the absence of circulation has lasted long enough to cause total and irreversible brain damage. Once it is believed that this damage has occurred (namely after the period of twenty minutes and thus the declaration of death as CD), physicians may initiate assisted circulation. Moreover, it is conducted in such a way as to prevent blood from reaching the upper part of the body and hence the brain (Ely 2022). However, this point is also considered debatable by those who emphasize that the absence of brain perfusion has never been proven, and there is reason to believe it might persist, thus making the practice of NRP ethically controversial (Entwistle et al. 2022). Unfortunately, a person already considered to be in BD in whose body blood is circulated by machines, cannot come back to life. The reason is that restoring brain circulation in BD patients is not associated with resumption of all brain functions.

Rather, the point to discuss is the “no touch period.” Indeed, if generally the duration of this period to declare a person’s death may not be deemed so significant, within the context of organ transplants it is crucial. In fact, the longer this duration, the higher the risk that the absence of circulation may damage the organs and render them unsuitable for transplantation. As we have noted, to address the limited availability of suitable organs relative to the rapidly growing demand, new strategies have been adopted to expand the unfortunately limited donor pool relative to the organ demand (Lomero et al. 2020), including the extension of donation criteria (Galeone et al. 2020; Zhang et al. 2020; Tian et al. 2020) and the adoption of DCD programmes in addition to DBD (DeVita, Snyder 1993; Lomero et al. 2020; Longnus et al. 2014; Antoine et al. 2014). However, the implementation of these DCD programmes can prove to be ineffective if the length of the no-touch period results in the organs being unsuitable for transplant due to damage incurred during that span of time. In the interest of enabling the procurement of unharmed organs, could it be considered ethically permissible to minimize or even disregard the no-touch period deemed sufficient to ensure BD?

In the case of Maastricht Category III, the organ donation occurs after a planned WLST. The two procedures (first the WLST and then the DCD) are typically kept separate to avoid the misunderstanding that WLST is performed to procure organs. However, we observe that the separation between WLST and DCD is purely formal. Indeed, evidence that the two procedures are closely linked is provided by the very existence of Maastricht Category III, which describes a “controlled” DCD where CD is expected following WLST, and after an “x” period of time organ procurement is initiated (Thuong et al. 2016).

The decision to perform a WLST can be made by physicians or by the patient (or by their loved ones in reconstructing their will). In the first case, the physicians deem the LST clinically inappropriate and decide to terminate them, based on the prohibition of futile care. This is the situation currently experienced in the clinical practice of Maastricht Category III DCD. Nonetheless, there are another instance where WLST may be pursued in a potential donor patient. This is the case of an individual who expresses informed dissent to continue LST either directly or through Advanced Directives, and the physicians, respecting the principle of self-determination, discontinue the treatments. The patient’s reasons may stem from the awareness that proceeding with treatments would unfortunately not restore their health, or from an individual inability to bear the burden of side effects or to face the risks associated with therapies relative to the expected benefits. However, the reasons for dissenting to continue with treatments might also not pertain to the interventions themselves and could instead be related to the person’s relational life or their existential perspective. Whatever the reason, in cases where the decision to withdraw from medical treatments is made by the individual, they are not obligated to share the reasons for their choice. Let’s consider the case where the patient wishes to communicate the reason to the physicians, and that reason is specifically to donate their vital organs. The result is that WLST will be executed specifically to facilitate DCD. According to some authors, patients requesting WLST should even be actively encouraged to donate (Jeong et al. 2021; Park et al. 2021). The patient’s self-determination thus translates both into controlling the timing and manner of their own death (Patuzzo et al. 2023) (not wanting to live in the clinical condition that involves LST) and in attributing a precise meaning to it (donating their organs). Indeed, if a donor requests WLST and explicitly tells the physician that the reason for requesting it is to die to donate their vital organs, the physician, to respect the patient’s autonomy, proceeds with the WLST and then harvests the organs. In this scenario, the physician pursues the patient’s good as subjectively defined by the patient themselves: to die in order to donate.

In any case, whenever a WLST is performed (regardless of who decides and why), the consequence is that the process of dying, which had been halted by medical treatments, resumes. In other terms, if the treatments had not been administered, the person would already be dead. On the other hand, we have mentioned that in Italy the objective of the no-touch period is that the absence of circulation leads to BD. Therefore, this waiting period loses its meaning in the case of WLST, as the outcome of this procedure is precisely the death of the individual (Zorko et al. 2023), otherwise we would not be discussing the cessation of LST. It can then be asserted that there is a distinct difference between allowing death to occur by waiting for a person to progress to CD and then BD after WLST, and actively killing by procuring vital organs immediately following WLST. We do not intend to delve into the specific debate between allowing to die and killing (Singer 1993; Asscher 2008), nor into the discussion distinguishing death from dying (Morison 1971; Kass 1971). However, we note that in the context of organ transplantation, the death of the person is not the sole ethical criterion to consider when evaluating the legitimacy of the organ procurement, and there exists the case of donors post-WLST.

In Maastricht Category III, there is an individual who wishes to altruistically donate their vital organs to save another person’s life (there is a donor) and who is in a condition where WLST is implemented, resulting in their inevitable death. In this context, waiting for the no-touch period post-CD to comply the DDR and with the concrete risk to damage the organs, means not honouring the other three ethical criteria for the mere minutes that separate the donor from an unavoidable death. Foregoing the no-touch period in Maastricht Category III entails an ethical evaluation of hastening the death of a donor destined to die to save others’ lives through organ donation. When physicians implement WLST on a donor patient, they do not do so with the intention of procuring organs but rather for previously mentioned reasons (to respect the prohibition against futile care or to honour the patient’s informed dissent, communicated directly or via Advance Directives, against continuing medical interventions). Once LST are discontinued, the individual enters a metaphorical tunnel at whose end, after a specified period, lies death. From this tunnel, there is no return, as the only means would be the reinstatement of LST, which we exclude since they have been discontinued for specific reasons. As mentioned, the individual in this tunnel intends to donate their organs, an end generally regarded as good since it aims to potentially save the lives of ill individuals who require such organs. Given this scenario, immediately after the WLST, we might consider shortening the duration of this tunnel by harvesting the vital organs to protect them. In recent years, the Italian Constitutional Court issued a ruling (No. 242/2019), which established that a patient with certain characteristics undergoing WLST can request MAS. The rationale of the ruling revolves around the idea of accelerating the death once LST have been discontinued, to respect the individual’s choice to avoid waiting the necessary period to die after WLST. Just as we might allow the acceleration of death post-WLST through MAS if the patient desires, we could also consider accelerating death after WLST through the harvesting of vital organs if the patient is a donor. If a patient is scheduled for WLST and is a donor, the individual’s good (the WLST and respect for their wish to donate) entails a benefit for society (the availability of organs suitable for transplantation).

The violation of the DDR in this context could thus be justified, theoretically by the non-absoluteness of the “Do not kill” prohibition and practically by considering it ethically good to pursue the will of a donor who is destined to die anyway. As we noted in the introduction, for the procedure of harvesting vital organs to be considered ethically permissible, four fundamental criteria are necessary: the donor must already be deceased, the purpose must be to protect health and save the lives of others, the decision to donate must be made freely, and the act must be a gratuitous act of solidarity towards others. In cases where LST are discontinued for a donor, the donor inevitably begins to die (we reasonably exclude the resumption of LST). It is within this inevitability that we might consider foregoing the first criterion in favour of the other three. In fact, what remains for the donor after WLST is to donate and, if this entails dying in a shorter time than what would have been naturally necessary after WLST, this does not automatically render ethically illicit the act of the one procuring that death by vital organs procurement. On the contrary, such an act could be interpreted as a moral duty of society to accept it and a moral duty of the physician to practice it. Indeed, the physician is called upon to pursue the patient’s good, which in this case is to donate in the face of an anticipated and inevitable death, for a common good, offering a tangible hope of continued life to those who can and still want to live.

## Conclusion

In the context of organ transplantation using DCD donors, organs could be damaged and become unsuitable for transplant during the no-touch period. As we have argued, waiting for this period after WLST to await the presumed occurrence of BD is contradictory since the consequence of WLST is precisely the patient’s death. Indeed, even though from a technical and clinical standpoint WLST is not performed to cause the patient’s death (but rather to avoid futility and/or respect the patient’s dissent to treatments), its outcome is effectively the death of the person. From the physicians’ perspective, their decision is made knowing that discontinuing the treatments will lead to the patient’s death. From the patient’s standpoint, if they have expressed their dissent with the continuation of the LST and are therefore asking for their withdrawal, they are fully aware that the cessation of LST will result in death, and consequently their wish to stop the medical treatments is in line with the desire to die. Therefore, to honour the patient’s wish to donate and to achieve the ethical objective of transplantation, in the context of DCD following WLST (Maastricht Category III), we should focus our efforts solely on the preservation of organs. Toward this end, we propose to forego the no-touch period, allowing physicians to proceed with the procurement of vital organs immediately after WLST. In the case of a donor for whom WLST has been planned, physicians should implement all necessary clinical measures so that DCD can be successfully carried out without wasting time after WLST.

Advancing the donor’s moment of death immediately after WLST does not equate to killing for the purpose of procuring their organs, nor does it imply a disrespect for their life. If we admit an exemption to the prohibition of killing (a person) under a specific condition (WLST) and for a specific purpose (organ transplantation), this exception would illustrate the reasonable possibility of breaching the ethical barrier between the dying process and organ procurement (the DDR) (Truog 2016), grounded on the consideration that following WLST the individual is no further harmable (Smith 2022) as they are destined to die and will die, and hence the other three ethical criteria (the willingness to donate altruistically to save other lives) should become our moral compass. Indeed, accelerating death after the WLST in a donor patient does not harm the dignity of the person or their memory, but rather benefits those who will have honoured their altruistic desire to donate, which likely aligns with their hope of not dying in vain.

In conclusion, in the case of a WLST on a donor patient, with the goal of respecting their wish to donate and thus preserve their organs for transplantation, and considering that the WLST will lead to their death, we might consider accelerating their death by harvesting the vital organs immediately after WLST. However, if we are prepared to admit this violation of the DDR, the ethical debate should also entertain the possibility of avoiding WLST altogether, allowing physicians to proceed directly with the organ extraction on the patient under anaesthesia, bypassing a withdrawal procedure that, while it might hold formal significance, would have lost its substantive meaning.

## Data Availability

Not applicable.
